# Does owner handedness influence paw preference in dogs?

**DOI:** 10.1007/s10071-022-01673-x

**Published:** 2022-09-03

**Authors:** Kimberley Charlton, Elisa Frasnelli

**Affiliations:** 1 School of Life Sciences, University of Lincoln, Lincoln, LN6 7DL Lincolnshire UK; 2 CIMeC Center for Mind/Brain Sciences, University of Trento, Piazza della Manifattura 1, 38068 Rovereto, TN Italy

**Keywords:** Handedness, Laterality, Dog, Behaviour, Paw preference

## Abstract

Handedness has proven to be the most effective and least intrusive measure of laterality in many species. Several studies have investigated paw preference in dogs (*Canis familiaris*) without considering the potential impact that owner’s handedness may have on it, despite dogs being a domesticated species. The aim of this study was to investigate whether owner handedness influences paw preference in their dogs. Sixty-two dogs had their paw preference tested using a Paw Task and a Reach Task in their home over 10 days, recorded by their owners. Interestingly, it was found that left-handed owners were more likely to own a dog with a left paw bias, and right-handed owners were more likely to own a dog with a right paw bias. In the Paw Task, the hand presented to a dog did not significantly predict which paw the dog lifted in response. Furthermore, it was found that females displayed a right paw bias at all age groups. However, males had a left paw bias in puppyhood and right paw bias in older age groups. We conclude that owner handedness influences paw preference in dogs, and it should be considered when suitably pairing dogs to potential owners, especially in assistance work.

**Supplementary Information:**

The online version contains supplementary material available at 10.1007/s10071-022-01673-x.

## Introduction

Handedness is the most common measure of behavioural lateralization in humans (Versace and Vallortigara 2015), with 90% of individuals being right hand dominant in most tasks (Papadatou-Pastou et al. 2020). Being right-handed is controlled by the left hemisphere of the brain, a crucial area for language and potentially accountable for the percentage of right-handed individuals among the human population (Corballis 2003). Lateralization is a feature of all mammalian species, as well as most vertebrates (Rogers 2002) and invertebrates (Frasnelli 2013; Rogers et al. 2013). In most vertebrate species, the right hemisphere has been linked to information processing and recognising conspecifics (Rogers 2002), with the left hemisphere used in discriminating between previously encountered objects (Rogers et al. 2004). Hand preference in non-human primates has been found to be task specific (McGrew and Marchant 1999), with complexity and animacy of an object affecting hand preference: A right-hand bias has been found only in behaviours directed towards inanimate target objects, but not towards animate targets (Fagot and Vauclair 1991; Forrester et al. 2011, 2012). Furthermore, the strength of laterality has been shown to be an indicator of emotional functioning in chicks and dogs with weak lateralization being more reactive in response to a stressor (Dharmaretnam and Rogers 2005; Branson and Rogers 2006). It was also found that left handedness is linked to showing stronger fear responses in marmosets (Braccini and Caine 2009). Left limb biases have also been linked to aggression, reactivity, and vigilance in numerous species (Schneider et al. 2013; Siniscalchi et al. 2014). As limb preference seems linked to emotional functioning (Leliveld et al. 2013; Versace and Vallortigara 2015) it could be a predictor of vulnerability and poor welfare. Therefore, limb preference could be a useful measure when considering the welfare of captive species (Gordon and Rogers 2015), and when considering the welfare of dogs in rescue centres and assistance dog training.

Handedness, or pawedness, is often assessed by observing the use of an animal’s dominant limb when participating in tasks (Batt et al. 2007). Paw preference in dogs has previously been studied through several tasks such as removal of tape on their nose (Quaranta et al. 2004), taking a first step (Simon et al. 2022; Tomkins et al. 2012; Barnard et al. 2017; Wells et al. 2018), retrieving food from a can (Wells 2003), lifting a paw (Wells 2003; Wells et al. 2018) or stabilising a stuffed Kong™ (Simon et al. 2022; Branson and Rogers 2006; Schneider et al. 2013; Siniscalchi et al. 2016). From a review of paw preference tests (Wells 2020), the two with the highest test–retest reliability and the easiest to implement for owners, without compromising welfare, are the reaching test (Wells 2003) and lifting a paw test (Wells 2003; Wells et al. 2018). Each of these tests independently involve either the owner or an interaction with an inanimate object. Using two paw preference tests per participant could, therefore, increase the validity of the data, as well as investigate the differences in the interactions.

Dogs show behavioural lateralization and have a population-level right paw preference in both male and female dogs. Dogs present a sex bias in pawedness, like human handedness, showing a right limb preference (Duncan et al. 2022), with a higher percentage of right pawedness in females than males (Hirnstein et al. 2019; Wells 2003; McGreevy et al. 2010; Quaranta et al. 2004; Laverack et al. 2021). It creates speculation among the field of other factors that could contribute towards what influences handedness, such as sex hormones. In contrast to this, a recent meta-analysis found no left or right paw bias at the population level, but only at the individual level in the species (Ocklenburg et al. 2019). Left paw laterality has been associated with an increased performance in dogs in tasks involving moving forward, searching, and finding objects (Van Alphen et al. 2005), whereas a weak paw preference makes a dog more likely to be distracted in agility training (Siniscalchi et al. 2014). Another factor found to influence paw preference in dogs is age, with older dogs showing a stronger right paw bias than younger dogs (Laverack et al. 2021). Dog’s pawedness is more defined, and therefore easier to identify, once the dog is closer to social maturity (Overall and Dunham 2002; Batt et al. 2008).

Although several genes have been associated with it (McManus et al. 2013), human handedness has been shown to be influenced by a number of environmental and cultural factors (e.g. Fagard and Dahmen 2004; Ocklenburg et al. 2010; Fagard et al. 2021), including parental influence (Laland 2008). Studies have proposed hypotheses on the role of human domestication in shaping dogs’ cognition, behaviour, and sociability (Range et al. 2019; Lazzaroni et al. 2020). As lateralization is expressed through behaviour and it is linked to cognition, human domestication may have played a role also in influencing dogs’ pawedness. Dogs have a similar ontogenetic experience to humans (Ocklenburg et al. 2019) and therefore, it can be argued that dogs can be used to delve deeper into human cognition (Johnston et al. 2017). Social learning has also played an integral part in the domestication process of dogs (Fugazza et al. 2018) and therefore it must be considered whether pawedness in dogs reflects their owners’ handedness. In agility-trained dogs, latency to complete obstacles was longer when owners were in the left visual field compared to the right (Siniscalchi et al. 2014). Thus, from lateralization studies with dogs, owners can gain more information to increase success in training and sports such as agility and flyball. Owner–dog compatibility is crucial in sporting success and strong lateralization has shown to be beneficial in improving the efficiency of behaviours (Rogers et al. 2013). Among other potential processes, it has been hypothesised that domesticated animals, like cats and dogs, may develop a stronger paw preference through imitation of humans (Ocklenburg et al. 2019). Social learning is advantageous for dogs as it reduces the risk of failure in trial-and-error learning and has proven to be a more efficient method of acquiring information (Fugazza et al. 2018) as well as setting them up for success. Although there is evidence of dogs learning through observing their owner (Kubinyi et al. 2003), behaviour that is repeated over time is more likely to be remembered. Therefore, it is important to consider the length of time a dog has been owned for as recently obtained dogs may not have ‘socially learned’ from their owners yet. Investigating the potential influence of owners on dog’s pawedness would expand knowledge on social learning and behaviour, as well as lateralization. This could optimise the success of training (Batt et al. 2008; Tomkins et al. 2010) as well as reduce unnecessary training and costs on potentially unsuitable dogs (Slabbert and Odendaal 1999). Paw preference tests have also proven to not negatively impact the dog’s welfare unlike other behaviour tests such as the Startle Test (Svatberg 2002) and the Strange Situation Test (adapted by Topál et al. 1998). Therefore, more research using paw preference tests will contribute to the research on their effectiveness and what they can show.

The aim of this study was to explore how owner’s handedness influences paw preference in dogs assessed by their owners through an online questionnaire and two tasks within their homes, a Paw Task (Wells et al. 2018) and a Reach Task (Wells 2003). These two tasks allowed us to obtain two measures of paw preference to assess the effect of interaction with the owner (in the Paw task) and with an object (in the Reach Task). The results of the two tasks were analysed against the information provided by the owners of their handedness; the dog’s age, neuter status, and sex. Given the premises of the potential effect of domestication and social learning, we could predict left-handed owners to be more likely to have left pawed dogs, and right-handed owners to be more likely to have right pawed dogs. Based on previous literature on paw preference in domestic dogs, we expected: (1) a predominant use of one paw, not necessarily the same, in both tasks; (2) a higher percentage of right pawed individuals in females than in male dogs; (3) older dogs will show a right paw preference more than younger dogs, especially males.

## Materials and methods

### Subjects

Sixty-two dogs of different breeds aged 0–12 participated in the study. Two owners specified that they were ambidextrous. Therefore, their data were removed. Of the remaining sixty dogs, 40 were Pedigree and 20 were Mixed Breed. Each dog was categorised into one of three age classes (Puppyhood, Adulthood, and Late Adulthood; Table 1; Wallis et al. 2016). There were 28 males (14 neutered, 14 unneutered), and 32 females (20 neutered, 12 unneutered). All the dogs were companion animals whose owners gave informed written consent to them participating in the study and acknowledged that they could not be identified via the study (see Supplementary Material). None of the subjects were reactive around toys, nor had any mobility problems which could create motor bias. Sixty different owners (50 right-handed and 10 left-handed) participated in the study. Participants were recruited through advertising on social media, and through the University of Lincoln’s Pets Can Do database.

**Table 1:** Age, sex, and neuter status of subjects

Life stage	Age in months	Female (neutered)	Male (neutered)
Puppyhood	0–24	8 (2)	7 (0)
Adulthood	> 24–72	13 (9)	14 (9)
Late adulthood	> 72	11 (9)	7 (5)
Total		32 (20)	28 (14)

### Questionnaire

The dogs’ owners were asked to complete a questionnaire using JISC online surveys prior to the paw preference tasks to gather information about themselves, their dogs, and their social interactions. The questionnaire provided information on the owner’s gender, handedness, and on the demographical data of their dogs: age, breed, sex, neuter status, and health (for details see the full questionnaire in Supplementary Material).

### Paw preference tests

Two previously employed tests were used and adapted to record the dogs’ paw preference: a Paw Task and a Reach task. All the dogs were required to undertake these two tasks within the dog’s homes over a 10 non-consecutive day period. To prevent carryover effects, the tasks were carried out at separate times during each day.

#### Paw Task

In the Paw Task, dogs were ensured to be sat symmetrically to prevent motor bias through uneven weight distribution. Owners were instructed to put one hand behind their back and offer their other hand flat upwards in front of their dog, keeping their arm central to their body. Following this, the owners would repeat the task using their opposite hand. Instructions were provided for the owners on which hand to present first each trial. The first hand presented to the dog alternated randomly each day, and each day consisted of two trials: one presenting the left hand, one presenting the right hand, and vice versa. The owner recorded the paw that was first lifted by the dog and ticked the LEFT or RIGHT box of the online questionnaire accordingly. If the dog did not lift a paw, the owner ticked the NIL box. This task was repeated with a maximum of two trials per day for the owner’s left and right hand for 10 non-consecutive days, generating a total of 20 paw lifts per animal for the Paw Task. (See Supplementary Material for the written instructions delivered to the owners with figures’ support).

#### Reach Task

A desired object, chosen by owners, was placed in an out-of-reach area that was small enough to prevent the animal retrieving the object with its mouth but large enough so the dog could comfortably put its paw underneath. If able to, the owners were instructed to ask their dog to sit and wait whilst the task was set up. If unable to, the dogs were to be gently held back by another person whilst the owner set up the study. The owner was instructed to place the desired object in the chosen area. They were then instructed to step away from the piece of furniture by approximately one metre to allow the dog to interact with the object. The owner recorded the paw that was first used by the dog to retrieve the object and ticked the LEFT or RIGHT box of the online questionnaire. If the dog did not interact with the object, or used their mouth, the owner ticked the NIL box. The trial was suspended after 60 s if there was no interaction to prevent frustration build-up. The owner remained present throughout the study but did not interact with the dog until the first paw used to reach for the object was recorded, or after 60 s. This task was repeated with a maximum of one trial per day for 10 non-consecutive days, generating a total of 10 reaches. The owner noted what object was used, and whether the object was consistent or changed throughout the study. (See Supplementary Material for the written instructions delivered to the owners with figures’ support).

### Analysis

All analyses were conducted in R 4.0.4 (R Core Development Team). To test the effects of owner handedness on dog’s paw preference in the two tasks, we fitted generalised linear mixed-effects models using the glmer function in the lme4 package (Bates et al. 2015). Handedness was calculated using a Laterality Index (LI) using the formula LI = (*L* − *R*)/*L* + *R*), where *L* and *R* indicate Left and Right pawed responses; therefore, a score of 1.0 represents exclusive left pawed use and − 1.0 represents exclusive right pawed use (Siniscalchi et al. 2008). LI was calculated separately for the Paw Task conducted by the owner presenting the Left and Right hand, and the Reach Task, providing three LI scores for each dog. When separating the left and the right hands presented for analysis, the tasks were referred to as Paw Task Left Hand and Paw Task Right Hand. Age group, sex, and neuter status (and their interactions) were fixed factors, and the unique reference number (URN) of the owner was a random effect to control for potential pseudoreplication.

## Results

**Fig. 1:**
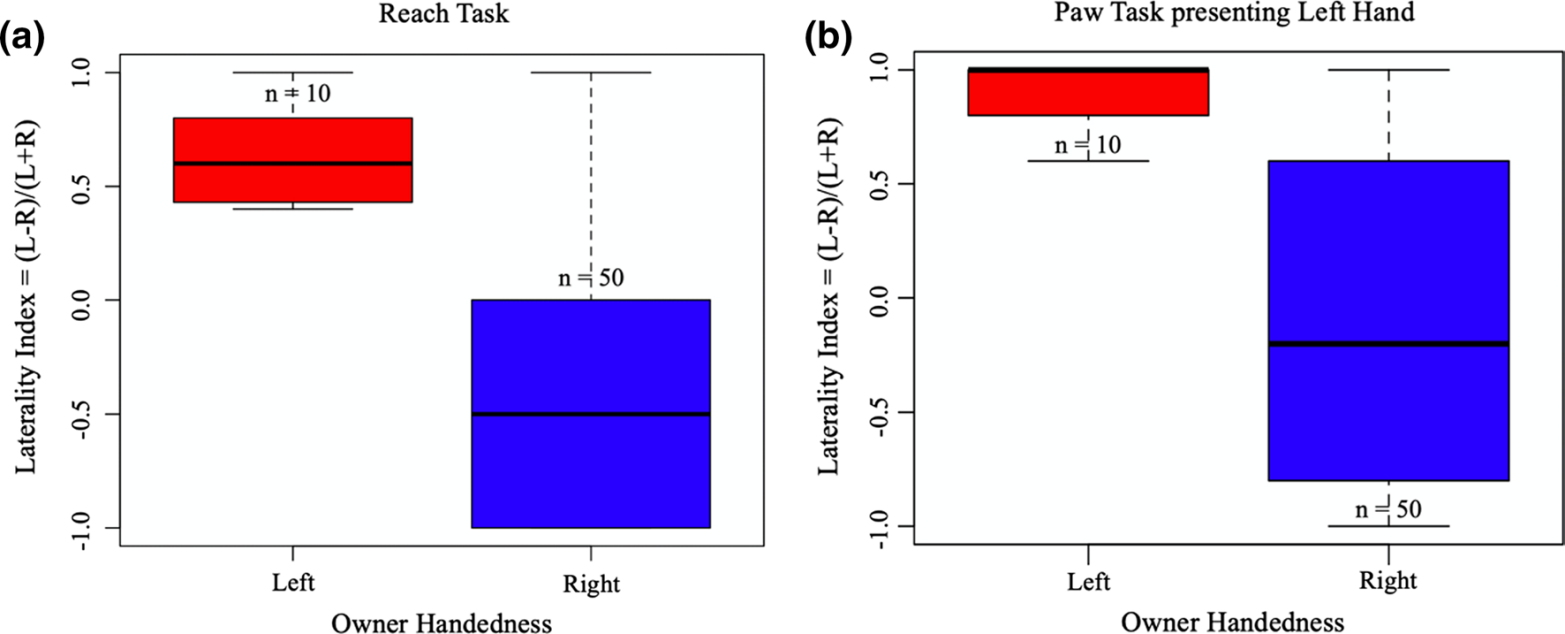
Median ± interquartile range of dogs’ pawedness in **a** the Reach Task and in **b** the Paw Task Left Hand for left- and right-handed owners

Following model simplification, the paw used in the Reach Task was significantly predicted by owner handedness (General Linear Model: *F*_1,54_ = 31.38, *P* < 0.001***; Fig. 1a). The results show a left paw bias in dogs owned by a left-handed owner, and a right paw bias in dogs owned by right-handed owners in the Reach Task. There was no significant effect of sex (*F*_1,54_ = 2.45, *P* = 0.12), age group (*F*_2,54_ = 1.18, *P* = 0.31) or neuter status (*F*_1,54_ = 0.42, *P* = 0.52) for the paw used in the Reach Task.

Following model simplification, the paw used in the Paw Task Left Hand was significantly predicted by owner handedness (General Linear Model: *F*_1,54_ = 16.72, *P* < 0.001***; Fig. 1b). This specifically shown left paw bias in dogs owned by a left-handed owner, and right paw bias in dogs owned by right-handed owners in the Paw Task Left Hand. There was no significant effect of sex (*F*_1,54_ = 0.24, *P* = 0.62), age group (*F*_2,54_ = 0.22, *P* = 0.80) or neuter status (*F*_1,54_ = 0.89, *P* = 0.35) for the paw used in the Paw Task Left Hand.

**Fig. 2:**
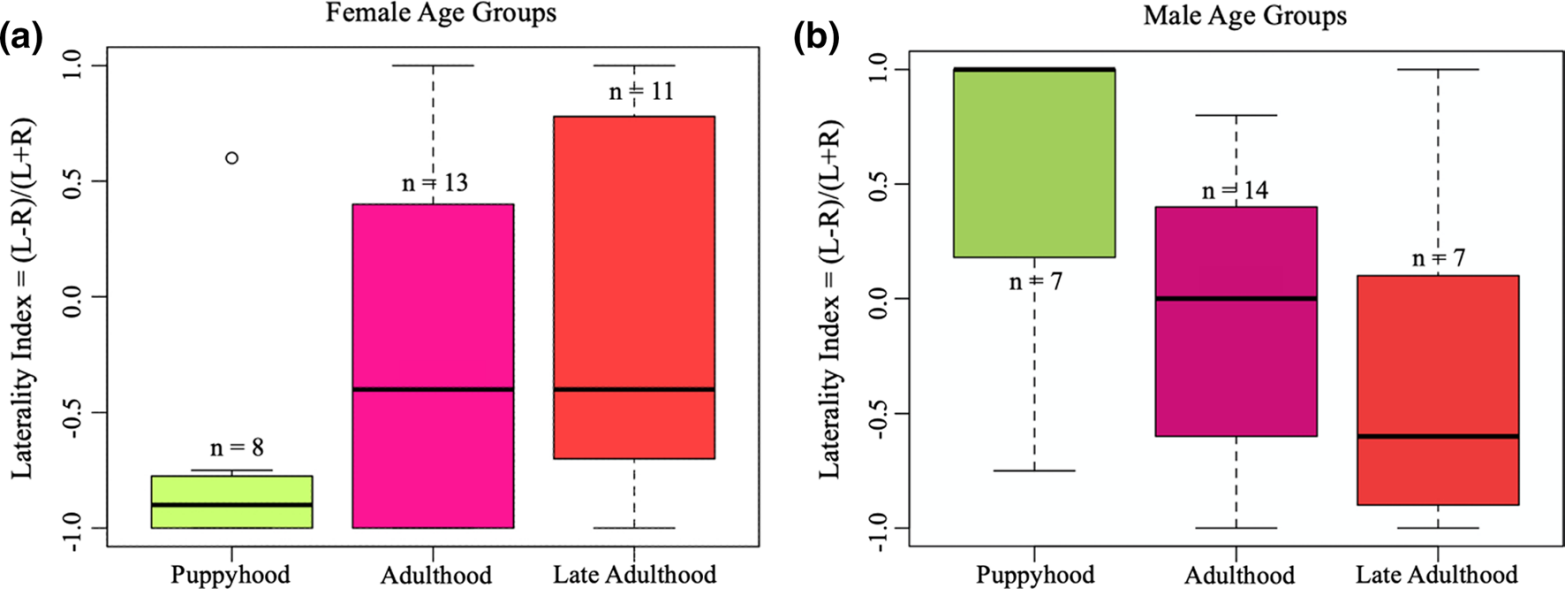
Median ± interquartile range of **a** female and **b** male dogs’ pawedness in the Paw Task Right Hand for the three age groups considered

Following model simplification, pawedness in the Paw Task Right Hand was significantly predicted by the interaction between sex and age group (General Linear Model: *F*_2,52_ = 4.38, *P* < 0.05*; Fig. 2a, b). Males showed a left paw bias in puppyhood, and a right paw bias in adulthood and late adulthood. In females, a right paw bias average was found in all three age groups. There was no significant effect of owner handedness (*F*_1,52_ = 1.27, *P* = 0.27), age group (*F*_2,52_ = 0.03, *P* = 0.97), sex (*F*_1,52_ = 2.47, *P* = 0.12) or neuter status (*F*_1,52_ = 0.13, *P* = 0.72) singly in the paw used in the Paw Task Right Hand. To have an 80% power of detecting a significance in owner handedness in the Paw Task Right Hand, a power analysis found *n* = 395 would be needed.

**Fig. 3:**
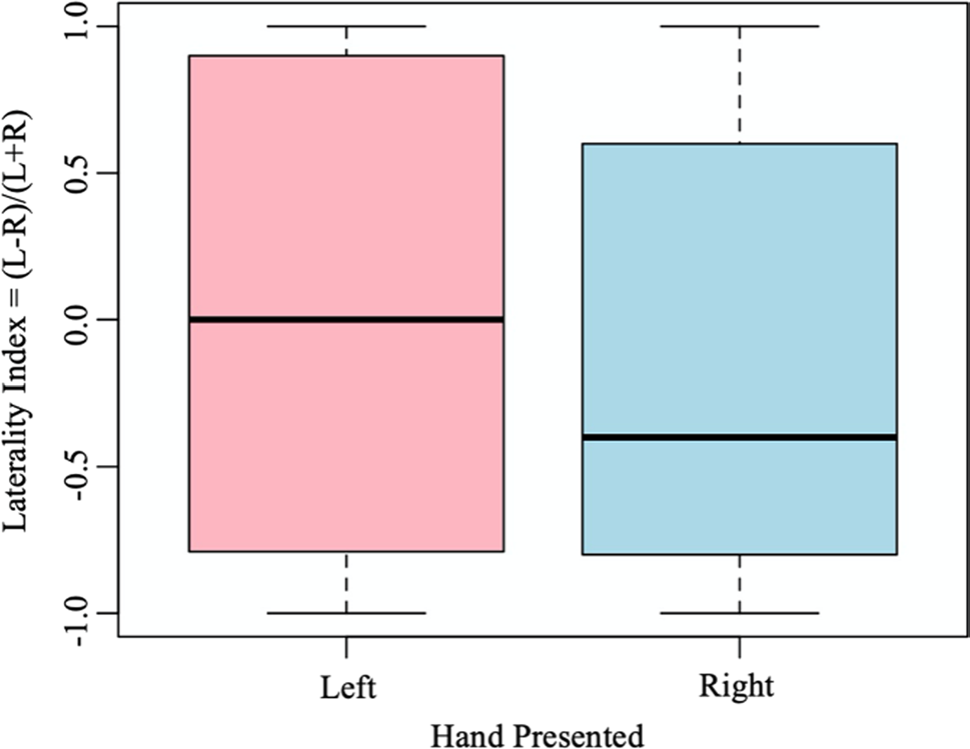
Median ± interquartile range of dog’s pawedness in the Paw Task for the left and right hand presented by the owners

After controlling for the pseudoreplication, the owner’s left or right hand presented to the dog in the Paw Task did not have a significant effect of the paw the dog lifted in response, although on average dogs preferred to lift their right paw in response to the right hand offered (General Linear Mixed-effects Model: *χ*^2^(1) = 3.62, *P* = 0.057; Fig. 3). Sex (*χ*^2^(1) = 1.61, *P* = 0.20), age group (*χ*^2^(2) = 0.04, *P* = 0.98) and neuter status (*χ*^2^(1) = 0.17, *P* = 0.68) did not have a significant effect of the paw the dog lifted in response to the hand presented.

## Discussion

The main aim of this study was to identify whether owner handedness influences paw preference in dogs. Left-handed owners were significantly more likely to have a dog with a left paw bias, and right-handed owners were significantly more likely to have a dog with a right paw bias. This was found in both the Reach Task and in the Paw Task Left Hand. Owner handedness was not shown to be a significant predictor of dog pawedness in the Paw Task Right Hand. The reason for this may rely in the—although not significant—tendency that dogs tended overall to give the right paw when the right hand was offered. However, the interaction of sex and age group was a significant predictor of paw use in the Paw Task Right Hand. In line with previous research, females shown a right paw bias more than males (Laverack et al. 2021). It was also found that older male dogs presented a right paw bias more than younger dogs, like previous research (Laverack et al. 2021). However, this was found to be more stable in females who generally displayed a right paw bias throughout all age groups, whereas male dogs averaged with a left paw bias in puppyhood but presented a right paw bias in adulthood and late adulthood. This could be due to sex hormones at different ages; however, neuter status was not found to have a significant effect on paw preference in dogs in either of the tasks. An additional plausible explanation of paw preference becoming more evident with age may be linked to experience: younger individuals may show a slight paw preference and, as they use more and more this paw, they become more skilled with it and the preference becomes stronger. However, this cannot explain alone the difference of age effect between female and male dogs.

In our study, owners were asked to test their dogs paw preference in two tasks, Paw Task (Wells 2003; Wells et al. 2018) and Reach Task (Wells 2003). These tasks have been reviewed for their efficacy in testing paw preference (Wells 2020). The Reach Task has previously proven to be more effective in cats than dogs (Wells 2020). However, it has shown to be an appropriate measure of paw preference in dogs in this study as all dogs completed the tasks (Duncan et al. 2022). By using two measures of paw preference, we were able to assess an interaction with the owner and with an object. The Paw Task involves owner participation; however, this was controlled for by asking the owners whether their dogs had been previously taught to ‘give paw’. Due to the high percentage of subjects that had been taught to ‘give paw’, i.e. 76.67%, it may be that the previous positive reinforcement for lifting a specific paw was more salient to the dogs than observing and imitating the hand the owners presented during the Paw Task. This could help explain the non-significance of paw lifted to hand presented. In comparison to this, the Reach Task aims to remove owner bias by having the dog interact with a desired object without the owner in peripheral view of their dog. However, we should also consider that, although owners were instructed “to move away from the furniture to reduce their presence causing bias in how the dog interacts” (see Supplementary Material) we could not prevent a possible influence of the owner movement and position during the task. For example, if the owners stepped away more to the right or left or were a bit to the left or to the right, instead of completely central, this may have biased what paw the dog used. This is especially true if we assume that typically dogs would have approached the object in a way that allowed them to have the maximum possibility of being in visual contact with the owner. Moreover, the significant result we found for the Reach Task could be a result of a higher ratio of right to left pawed dogs (Laverack et al. 2021), in comparison with the higher rate of right-handedness in humans (Papadatou-Pastou et al. 2020). Nevertheless, our data show that owner handedness significantly predicts dog pawedness.

This study is based on data entirely collected by the dogs’ caregivers and therefore lacks the scientific precision of a laboratory experiment as the researchers could not control for extraneous variables. However, owners were given the option to score ‘NIL’ if their dog did not present a paw at any time during the tasks and we see no reason as to why the owners would provide incorrect information, or why they would feel the need to produce biased answers. Furthermore, there were several ‘NIL’ entries sporadically throughout the trial days, which shows that the owners felt they could enter the data accurately and did not receive pressure to select Left or Right accordingly. As well as detailed descriptions, photographs were provided to inform the human participants what was needed in the study and how to set up the tasks to ensure the study was as valid as possible in a home environment, and to ensure the study would be replicable for further research. Nevertheless, we cannot exclude that some caregivers may have committed small mistakes (e.g. in their position relative to the dog) which may have affected the results as an experimental setup like the one used in this study requires such precision and symmetry. The study took place within the owner’s and dog’s home environment encouraging natural behaviour for the dogs and increasing the validity of data. However, in the future it would be important to run the study in a controlled experimental setup and also, as discussed above, to use an experimenter in addition to the caregiver to investigate this topic further.

There were more left-handed participants in the study when compared to the general population discovered in previous research (Papadatou-Pastou et al. 2020), with 17% of the owners reporting to be left-handed. However, due to the nature of the study, it may appeal to left-handers to participate in a study as being left-handed is not considered ‘the norm’. Owner handedness was a highly significant predictor of paw preference in the Reach Task, and a significant predictor in the Paw Task Left Hand. This supports our hypothesis that owner handedness influences paw preference in dogs. Through development in the human environment, dogs have become socialised and learn through trial-and-error learning (Fugazza et al. 2018) and may have developed a stronger paw preference through their imitation of humans (Ocklenburg et al. 2019). In the Paw Task, if dogs have been previously positively reinforced to lift a specific paw in response to their owner’s left or right hand, then they will be more inclined to repeat this behaviour (Wells 2020). Therefore, it must be considered whether the Paw Task alone is a true reflection of the dog’s paw preference, which supports the use of two paw preference tests in this study. Although on average dogs preferred to give their right paw when the right hand was offered, the owner’s hand presented in the Paw Task did not have a significant effect on the paw lifted. Thus, this may suggest a longevity in the paw preference learned through the imitation of humans (Ocklenburg et al. 2019) and their handedness. Recent work has challenged the real presence or absence of imitation as a form of learning in the canine species (Huber et al. 2020). Indeed, in their study, Huber et al. (2020) showed that dogs develop such a strong relationship with their caregivers and are so eager to learn from them that they copy even causally irrelevant actions. Interestingly, the same pattern of “overimitation” for unnecessary actions does not occur when the actions are demonstrated by an experimenter instead of the caregiver (Huber et al. 2020). To verify this, in the future, it would be interesting to replicate our experiment with an experimenter instead of the caregiver.

For the non-significant result of owner handedness in the Paw Task Right Hand, a power analysis was undertaken and a recommended sample size for a significant result is 395. The subject size for this study, although small in comparison to previous studies (Laverack et al. 2021), was representative of mixed breed and pedigrees, including a subject from each breed group. The subjects were of various ages with a balance of females to males as well as a mix of neutered and unneutered. Therefore, the data are representative of dogs owned in the UK and can be generalised accordingly.

The interaction of sex and age was significantly associated with paw preference. For females, an average right paw bias was found consistent among all age groups, whereas males averaged with a left paw bias in puppyhood but stabilised with an average right paw bias in the older age groups. This is in line with previous studies (Laverack et al. 2021). The development in male hormones could be a factor in the change of paw preference through ages; however, neuter status was not found to be a predictor of paw preference nor in this study nor in previous research (Duncan et al. 2022) so future research should investigate which developmental factors may influence paw preference in male dogs.

For further research, it is important to consider how long a dog has been owned for before owner handedness influences the dog’s paw preference. The survey questioned when the dogs were obtained; however, it was difficult to work out the amount of time each dog was owned due to the age categories used in the questionnaire; and estimating would have decreased the validity of the analysis. In our study, we classified dogs into three age groups due to the constraint of 60 subjects, but the categories consisted of a wide age range. Therefore, to pinpoint when a dog’s paw preference stabilises, a shorter age range would be necessary. Further research into the critical periods of paw preference would not only increases our understanding of dog’s cognition (Range et al. 2019; Lazzaroni et al. 2020), but would improve training in assistance work (Batt et al. 2008; Tomkins et al. 2010) by ensuring the training is undertaken at critical periods.

Paw preference could be used to assess the suitability of an animal for assistance work (Wells 2020). If owner handedness has an influence on dog’s pawedness, it needs to be considered whether this could impact the human–canine relationship (e.g. through better coordination in some tasks—Ghirlanda et al. 2009), and whether it could determine a dog’s success within training or participating in a sport. Motor lateralization, or pawedness, has an association in the success of guide dog training (Batt et al. 2008; Tomkins et al. 2010). Therefore, further research into the area could optimise success of training whilst also suitably pairing a guide dog with its compatible left-, or right-handed owner. Research into this topic could also improve success for rehoming dogs. Paw preference tests are not only effective, but they do also not negatively impact the dog’s welfare and incite stress unlike other behaviour tests such as the Startle Test (Svartberg 2002) and the Strange Situation Test (adapted by Topál et al. 1998). If more research could support the importance of paw preference tests to assess suitability of assistance work, it could reduce the use of less animal welfare friendly tests whilst continuing to save unnecessary training and costs on potentially unsuccessful, or unsuitable, dogs (Slabbert and Odendaal 1999). Furthermore, if owner handedness is found to have an influence on dog’s pawedness, then it produces more opportunity for the dogs to continue assistance work with further training or sets up the next owner for success by having a compatible pawedness–handedness.

In conclusion, our data suggest that owner handedness influences paw preference in dogs, although we need to consider the limits of the current study in terms of data collection. There is also strong evidence of a sex and age interaction influencing strength and direction of paw preference in dogs. This highlights the importance of further research into dog’s sex and age hormones that could contribute towards paw preference. We believe this study provides an important starting point for future research on the influence of caregivers’ handedness on dogs’ paw preference and potentially on the efficiency of assistance dog training to match dogs with potential owners more suitably.

## Supplementary Information

Below is the link to the electronic supplementary material.

Supplementary file1 (XLSX 33 KB)

Supplementary file2 (DOCX 1061 KB)
