# Supplementary material for: Does owner handedness influence paw preference in dogs?
Source: Anim Cogn. 2022 Sep 3;26(2):425–33. doi: 10.1007/s10071-022-01673-x (PMC9950156; doi:10.1007/s10071-022-01673-x)
Supplement: Supplementary file 2 — Supplementary file2 (DOCX 1061 KB) [file 10071_2022_1673_MOESM2_ESM.docx]

Supplementary Material

Participant Information Sheet/Information about the research

**Title of study:**Does owner’s handedness influence paw preference in dogs?

**Name of researchers:**Kimberley C. Charlton, Dr Elisa Frasnelli

**Contact details of the researchers are given at the end.**

We would like to invite you to take part in our research study. Joining the study is entirely up to you. Before you decide, we would like you to understand why the research is being done and what it would involve for you. In this study, we are investigating whether handedness affects paw preference in dogs. For this purpose, we have designed 2 different tasks with your dog. Both tasks can be easily done at home. In addition to a willing pet dog, an object such as a toy and some tasty dog treats, you will need a piece of furniture such as a sofa, risen bed, or sideboard to conduct the task.

You have **28 days to complete**the work if you wish to start it. You can start at any time. It is important that you always use the same device (e.g., computer or smartphone) to **give your results online.**This way you do not need to log in, you simply click on the link.

Before you decide to participate in this study, please read the following information sheet carefully. If you need further information, please contact us (details given at the end of this sheet).

**What is the purpose of the study?**

The purpose of the study is to investigate whether owner handedness influences paw preference in dogs. We are looking at your dog’s paw preference when engaging with you, and when interacting with an inanimate object. We will also consider other factors such as your handedness, what side of you your dog walks on and which hand you use to hold the lead.

**Am I eligible to take part?**

Anyone aged 18 years and above who has a dog with no medical/health conditions that affect their mobility and ability to bear weight on three legs for a short period of time can take part. Dogs who guard resources such as food or toys should also not participate in this study.

You are being invited to take part because you are above 18 years of age and own a dog. We are inviting 70 participants like you to take part.

**Do I have to take part?**

Participation is completely voluntary. You can withdraw at any time from this study without giving any reasons. This will not affect your legal rights. You should only take part if you want to and choosing not to take part will not disadvantage you in anyway.

**What will I be asked to do?**

If you want to take part in our study, you will be asked to complete an eConsent form. You will then be asked to conduct two tasks with your dog that you can easily do at home. In order to do the task, you may need some common household items (listed above). Ideally, the test should be carried out when you are alone with your pet at a time when you will not be disturbed. Detailed instructions and photos of how to do these will be provided. The test should fun for you and your pet and bring a little variety to your daily routine. The test takes between 5-10 minutes during the days you wish to do the trials. In total, over the 10 trial days, the test may take you about**50-100 minutes**.

Apart from the test, we will ask you some general questions about your dog (e.g., age, breed, neuter status).

You will have **28 days to complete** the work and send us the results online. Within this time, you can always get back to your survey by simply clicking on the link again! **If you want to get back to your survey, please note that you must use the same device and web browser and do not delete any cookies on your system.**
*Optional*: If you wish, you can upload videos taken of your dog during the test (a link for uploading videos will be provided). In case you would like to share your videos, please make sure that single videos do not exceed 50mb and that no sensitive data is visible in the videos that could identify you. If videos contain any sensitive information, they will be deleted immediately and cannot be used for data analysis. You will be given a unique ID subsequently to this introduction. When uploading the videos please make sure that the video name is in the following format: **ID_TestName_DAY_TrialNumber** (example:123456_PawTask_2407_Trial1).

**Will I be paid expenses for taking part?**

You will not be paid to participate in the study.

**What are the possible benefits of taking part?**

The test should be fun for both you and your dog and can add variety to your everyday life under the current circumstances of COVID-19.

**What are the possible disadvantages and risks of taking part?**

We cannot foresee any risks in testing your dog at home. However, you can always withdraw from this study if you are concerned about anything.

**Will anyone know I have taken part?**

The information we collect will be handled in confidence. No one will know you have taken part.

**Where will my data be stored?**

The data obtained from the study will be stored securely on the university OneDrive in password protected files. Only the researcher/researchers will have access to it. Paper copies will be stored in a secure cabinet/office at the University. The data from this study *may*be put in an Open Access repository for other researchers to use in future research. If so, responses will be anonymised and any personal data (e.g. contact details) will be removed.

If you decide to upload video files, you will be given a unique ID number to name your files. This will maintain your anonymity. If you want to delete your videos after you have already uploaded them, please contact us with your ID number so that we can delete the data. Please note that a subsequent deletion of your videos is limited to the duration of this study, as all data is completely anonymized for statistical analysis so that it is then no longer possible for us to identify your data.

**What will happen if I don’t want to carry on with the study?**

You are free to withdraw at any point from this study, without having to give a reason, by contacting us on either of the emails stated below.

If you choose to withdraw from the study, the information you have given us up to this point will be deleted/destroyed. However, once the anonymised data set has been created it will not be possible to remove your anonymised data from the analysis.

**What will happen to the results of the research study?**

The data collected in the questionnaire will be analysed in such a way that they cannot be assigned to individual participants. The results form the basis for a master's thesis and, after analysis, a summary of this work will be published via social media, e.g. Twitter (@UoLLifeSciences) or Facebook (UoLLifeSciences).

**Who is organising and funding the research?**

This research is being conducted by the University of Lincoln.

**Who has reviewed the study?**

All research conducted by the University of Lincoln is looked at by an independent group of people, called a Research Ethics Committee, to protect your interests.

**What if there is a problem?**

If you have a concern about any aspect of this study, you should ask to speak to the researchers who will do their best to answer your questions. The researchers contact details are given at the end of this information sheet. If you remain unhappy and wish to complain formally, you can do this by contacting [ethics@lincoln.ac.uk](mailto:ethics@lincoln.ac.uk).

**Further information and contact details**

Kimberley C Charlton, [25227855@students.lincoln.ac.uk](mailto:25227855@students.lincoln.ac.uk)

Dr Elisa Frasnelli, [efrasnelli@lincoln.ac.uk](mailto:efrasnelli@lincoln.ac.uk)

Information compliance

The University of Lincoln is the lead organisation for this study and will be the data controller for this study. This means that we are responsible for looking after your information and using it properly.

The university’s **Research Participant Privacy Notice** (<https://ethics.lincoln.ac.uk/research-privacy-notice/)> explains how we will be using information from you in order to undertake this study.

If you feel that we have let you down in relation to your information rights then please contact the Information Compliance Team by email on [compliance@lincoln.ac.uk](mailto:compliance@lincoln.ac.uk) or by post at Information Compliance, Secretariat, University of Lincoln, Brayford Pool, Lincoln, LN6 7TS.

You can also make complaints directly to the Information Commissioner’s Office (ICO). The ICO is the independent authority upholding information rights for the UK. Their website is ico.org.uk and their telephone helpline number is 0303 123 1113.

Top of Form

p. 2 Consent Form

Top of Form

Bottom of Form

**Title of Project: Does owner’s handedness influence paw preference in dogs?**

**Name of Researchers: Kimberley Charlotte Charlton, Dr Elisa Frasnelli**

Top of Form

1 1. I confirm that I have read the information sheet dated 09/04/2021 for the above study. I have had the opportunity to consider the information, ask questions and have had these answered satisfactorily.

Top of Form

- Yes
- No

2 2. I understand that should I withdraw then the information I have given us up to this point will be deleted/destroyed. However, once the anonymised data set has been created it will not be possible to remove my anonymised data from the analysis.

Top of Form

- Yes
- No

3 3. I understand that individuals from the University of Lincoln may look at research data collected during the study, to ensure that the study is conducted appropriately. I give permission for these individuals to have access to my records; I understand that my personal details shall be kept confidential.

Top of Form

- Yes
- No

4 4. I understand that the information collected about me will be used to support other research in the future and may be shared anonymously with other researchers.

- Yes

Top of Form

- No

5 5. I would like to receive a summary of the results of the study.

Top of Form

- Yes
- No

6 6. I agree to take part in the above study.

Top of Form

- Yes
- No

*If ‘No’ is selected on questions 1, 2, 3, 4 and 6 then the participant will be redirected to the end of survey page*

p. 3 Questionnaire about you

Top of Form

7 Participant email address:

Top of Form

8 Are you 18 years old or over?

Top of Form

- Yes
- No

*If ‘No’ is selected for question 8 then the participant will be redirected to the end of survey page as they are unsuitable for the study*

9 What gender do you identify with?

Top of Form

- Male
- Female
- Prefer not to answer
- Other

a (*If ‘Other’ is selected*) Please specify:

Top of Form

10 What hand do you predominantly use?

Top of Form

- Left
- Right
- Both/Ambidextrous

11 How many people are in your household in total?

Top of Form

- Just me
- 2 people
- 3 people
- 4 people
- 5+ people

12 How many dogs do you own?

Top of Form

- 1
- 2
- 3
- 4
- 5+

You are welcome to carry out this study on multiple dogs in your household. If you wish to do this, please complete the questionnaire and study for your chosen dog. Once the study has been completed, you will be given the choice to complete the study for another dog in your household if you wish.

**Please answer the following questions and complete the study with** **your chosen dog**

Top of Form

13 Who spends the most time with your dog?

Top of Form

- Me
- A relative
- My friend
- My partner
- A dog walker
- Other

a (*If ‘Other’ is selected*) Please specify:

Top of Form

b In relation to the person who spends the most time with your dog - what hand do they predominantly use?

Top of Form

- Left
- Right
- Both/Ambidextrous

***Please refer to the pictures below for guidance on the next two questions***

Top of Form

14 When walking your dog on the lead, where is your dog most likely to be? If this depends on which side of the road you are walking on, consider this question when you are in an environment such as a park

Top of Form

- On my left
- On my right
- In front of me
- Behind me
- In front of me, on my left
- In front of me, on my right
- Behind me, on my left
- Behind me, on my right
- Not sure
- Not applicable

a Which hand do you predominantly use when holding the lead?

Top of Form

- Left hand
- Right hand
- Both/Either


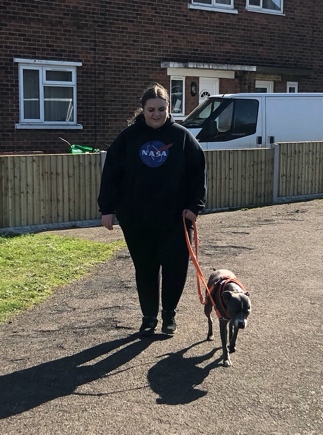

Example of dog walking on the left of person holding the lead with their left hand


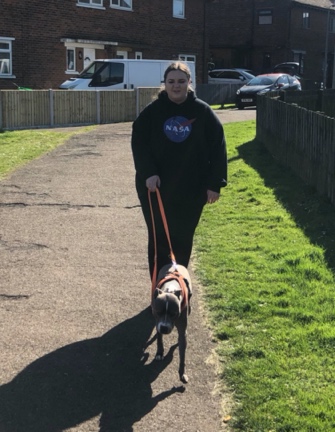
 


Example of dog walking in front of person holding the lead with their right hand


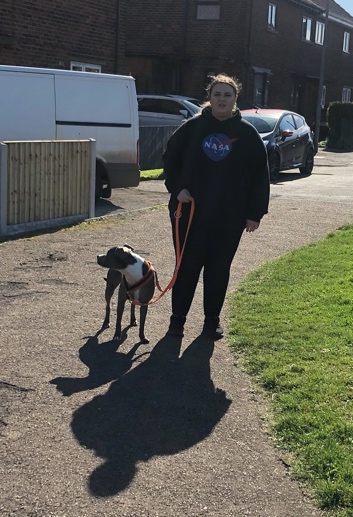

Example of dog walking on the right of person holding the lead with their right hand

Top of Form

p. 4 Questionnaire about your dog

Top of Form

15 How old is your dog?

Top of Form

- 0 - 6 months
- 6 months - 1 year
- 1 - 2 years
- 2 - 3 years
- 3 - 4 years
- 4 - 5 years
- 5 - 6 years
- 6 - 7 years
- 7 - 8 years
- 8 - 9 years
- 9 - 10 years
- 10 - 11 years
- 11 - 12 years
- 12 years+

16 Age of dog when obtained:

Top of Form

17 Where did you source your dog from?

Top of Form

- Family/Friend
- Private breeder
- Shelter/Rescue centre
- Bred myself
- Found
- Other

a (*If ‘Other’ is selected)* Please specify:

Top of Form

18 Is your dog Pedigree or Mixed Breed?

Top of Form

- Pedigree
- Mixed Breed

a (*If ‘Pedigree is selected*) What breed group is your dog?

Top of Form

Bottom of Form

Question actions

- Hound
- Working
- Terrier
- Gundog
- Pastoral
- Utility
- Toy

i (*If ‘Toy’ is selected - box opens up to display all Toy breeds*) Please specify:

Top of Form

- Affenpinscher
- Australian Silky Terrier
- Bichon Frise

Show all (23)

ii *(If ‘Utility’ is selected - box opens up to display all Utility breeds*) Please specify:

Top of Form

- Boston Terrier
- Bulldog

Show all (24)

iii (*If ‘Pastoral’ is selected - box opens up to display all Pastoral breeds)* Please specify:

Top of Form

- Anatolian Shepherd Dog
- Australian Cattle Dog
- Australian Shepherd

Show all (32)

iv (*If ‘Gundog’ is selected - box opens up to display all Gundog breeds*) Please specify:

Top of Form

- Bracco Italiano
- Brittany
- English Setter

Show all (32)

v *(If ‘Terrier’ is selected - box opens up to display all Terrier breeds)* Please specify:

Top of Form

- Airedale Terrier
- Australian Terrier
- Bedlington Terrier

Show all (26)

vi *(If ‘Working’ is selected - box opens up to display all Working breeds*) Please specify:

Top of Form

- Alaskan Malamute
- Beauceron
- Bernese Mountain Dog

Show all (24)

vii *(If ‘Hound’ is selected - box opens up to display all Hound breeds*) Please specify:

Top of Form

- Afghan Hound
- Basenji
- Basset Bleu De Gascogne

Show all (34)

19 Sex of dog:

Top of Form

- Male
- Female

20 Neuter status:

Top of Form

- Neutered
- Entire

a (If ‘Neutered’ is selected) Age at which the dog was neutered:

Top of Form

p. 5 Dog's Health Questionnaire

Top of Form

*WARNING:*

*The research involves a task which requires your dog being able to lift their paws, bearing weight on three limbs. The research also involves your dog reaching for an object, so please consider neck/back conditions in case of strain to these areas. The study is to be undertaken over 10 days so please be mindful of your dog repeating these tasks for 10 consecutive days.*

Top of Form

Bottom of Form

21 Does your dog have any health conditions which could affect the movement of their legs? (This could include, but is not limited to, arthritis, hip dysplasia, broken limbs, sprains etc.)

Top of Form

- Yes
- No

a (*If ‘Yes’ is selected*) Please specify:

*If ‘Yes’ is selected, then the participants will be redirected to the end of survey page as they are unsuitable for the study*

Top of Form

22 Does your dog have any other medical conditions?

Top of Form

- Yes
- No

a (*If ‘Yes’ is selected*) Please specify:

Top of Form

23 Does your dog take any medication?

Top of Form

- Yes
- No

a (*If ‘Yes’ is selected*) Please specify what medication, dosage and reason for taking this medication:

Top of Form

p. 6 Reactiveness Questionnaire

Top of Form

*WARNING:*

*This research requires a desired object being placed out of reach of the dog. This may build frustration in your dog. If there is a risk of your dog becoming reactive around treats/toys then please do not take part in the study*

Top of Form

24 Does your dog become aggressive/reactive around food or toys?

Top of Form

- Yes - around food only
- Yes - around toys only
- Yes - around both food and toys
- No

a (*If ‘Yes’ is selected*) Please specify how your dog reacts:

If ‘Yes - around toys only’ or ‘Yes - around both food and toys’ is selected then the participant will be redirected to the end of survey page as they are unsuitable for the study

Top of Form

p. 7 Previous training

Top of Form

25 Has your dog been previously trained, by you or another person, to 'give paw'? Give paw definition - Trained to lift a paw when a person puts their hand out and put the paw in the person’s hand

Top of Form

- Yes
- No
- Unsure

p. 8 Task One - Paw Task

Top of Form

For this research, we will look at which paw is more dominant through two tasks and compare whether the dog uses the same paw for each task, as well as looking at similarities in paw preference when interacting with an animate (person) and inanimate object (i.e., toy).

**Once you have completed Day 1 of Paw Task, click 'NEXT' to complete Day 1 of Reach Task. When you have completed your two tasks for that day, select 'FINISH LATER' and follow the instructions so you can return to the study and complete subsequent task days without losing any of your previous answers**

Top of Form

**Task One - Paw Task**

In task one, you should place one hand behind your back, and offer your other hand flat upwards in front of your dog. Your arm should be kept **as central to your body as possible**(as shown in the Figures below). Allow your dog time to respond, and record which paw, if any, they lift first.

You will then put that hand behind your back and offer your dog your other hand, again central to the body and flat facing upwards. Please try refrain from giving your dog a verbal cue. Record which paw, if any, your dog lifts first.

This task should be repeated 20 times (10 offering your left hand as shown in Figure 4., 10 offering your right hand as in Figure 5.) as listed in the table below and should only be repeated twice a day maximum.

You may notice in the table the hand that is presented first may alternate each time, this is to randomise it and control unwanted biases. This task can be undertaken any time over a 28 day period when it is convenient for you and your dog. The task will take 10 days in total. Once the task has been undertaken 10 times, no further trials are needed. Please complete the tables below. Follow the order of the table when presenting your left or right hand and record whether your dog gave their left (Left) or right (Right) paw, or if they did not lift a paw (NIL).


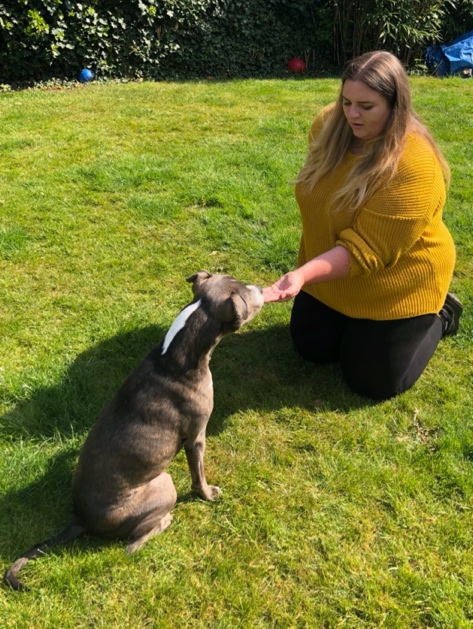

Example of person presenting their left hand to dog in Paw Task


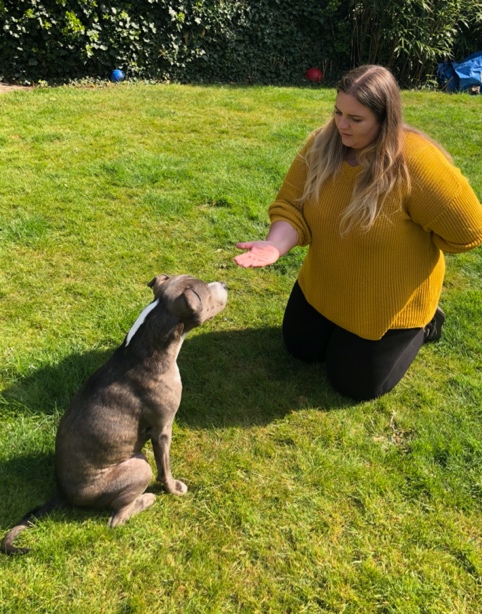

Example of person presenting their right hand to dog in Paw Task

Top of Form

26 Day 1

Top of Form

| Top of Form  Bottom of Form | Top of Form  Bottom of Form  Paw given | | |  |
| --- | --- | --- | --- | --- |
|  | Left | Right | NIL |  |
| Right hand offered | Radio button | Radio button | Radio button |  |

a

Top of Form

| Top of Form  Bottom of Form | Top of Form  Bottom of Form  Paw given | | |  |
| --- | --- | --- | --- | --- |
|  | Left | Right | NIL |  |
| Left hand offered | Radio button | Radio button | Radio button |  |

27 Day 2

Top of Form

| Top of Form  Bottom of Form | Top of Form  Bottom of Form  Paw given | | |  |
| --- | --- | --- | --- | --- |
|  | Left | Right | NIL |  |
| Left hand offered | Radio button | Radio button | Radio button |  |

a

Top of Form

| Top of Form  Bottom of Form | Top of Form  Bottom of Form  Paw given | | |  |
| --- | --- | --- | --- | --- |
|  | Left | Right | NIL |  |
| Right hand offered | Radio button | Radio button | Radio button |  |

28 Day 3

Top of Form

Bottom of Form

| Top of Form  Bottom of Form | Top of Form  Bottom of Form  Paw given | | |  |
| --- | --- | --- | --- | --- |
|  | Left | Right | NIL |  |
| Left hand offered | Radio button | Radio button | Radio button |  |

a

Top of Form

| Top of Form  Bottom of Form | Top of Form  Bottom of Form  Paw given | | |  |
| --- | --- | --- | --- | --- |
|  | Left | Right | NIL |  |
| Right hand offered | Radio button | Radio button | Radio button |  |

29 Day 4

Top of Form

| Top of Form  Bottom of Form | Top of Form  Bottom of Form  Paw given | | |  |
| --- | --- | --- | --- | --- |
|  | Left | Right | NIL |  |
| Right hand offered | Radio button | Radio button | Radio button |  |

a

Top of Form

| Top of Form  Bottom of Form | Top of Form  Bottom of Form  Paw given | | |  |
| --- | --- | --- | --- | --- |
|  | Left | Right | NIL |  |
| Left hand offered | Radio button | Radio button | Radio button |  |

30 Day 5

Top of Form

| Top of Form  Bottom of Form | Top of Form  Bottom of Form  Paw given | | |  |
| --- | --- | --- | --- | --- |
|  | Left | Right | NIL |  |
| Left hand offered | Radio button | Radio button | Radio button |  |

a

Top of Form

| Top of Form  Bottom of Form | Top of Form  Bottom of Form  Paw given | | |  |
| --- | --- | --- | --- | --- |
|  | Left | Right | NIL |  |
| Right hand offered | Radio button | Radio button | Radio button |  |

31 Day 6

Top of Form

| Top of Form  Bottom of Form | Top of Form  Bottom of Form  Paw given | | |  |
| --- | --- | --- | --- | --- |
|  | Left | Right | NIL |  |
| Right hand offered | Radio button | Radio button | Radio button |  |

a

Top of Form

| Top of Form  Bottom of Form | Top of Form  Bottom of Form  Paw given | | |  |
| --- | --- | --- | --- | --- |
|  | Left | Right | NIL |  |
| Left hand offered | Radio button | Radio button | Radio button |  |

32 Day 7

Top of Form

| Top of Form  Bottom of Form | Top of Form  Bottom of Form  Paw given | | |  |
| --- | --- | --- | --- | --- |
|  | Left | Right | NIL |  |
| Right hand offered | Radio button | Radio button | Radio button |  |

a

Top of Form

| Top of Form  Bottom of Form | Top of Form  Bottom of Form  Paw given | | |  |
| --- | --- | --- | --- | --- |
|  | Left | Right | NIL |  |
| Left hand offered | Radio button | Radio button | Radio button |  |

Top of Form

33 Day 8

Top of Form

| Top of Form  Bottom of Form | Top of Form  Bottom of Form  Paw given | | |  |
| --- | --- | --- | --- | --- |
|  | Left | Right | NIL |  |
| Left hand offered | Radio button | Radio button | Radio button |  |

a

Top of Form

| Top of Form  Bottom of Form | Top of Form  Bottom of Form  Paw given | | |  |
| --- | --- | --- | --- | --- |
|  | Left | Right | NIL |  |
| Right hand offered | Radio button | Radio button | Radio button |  |

34 Day 9

Top of Form

| Top of Form  Bottom of Form | Top of Form  Bottom of Form  Paw given | | |  |
| --- | --- | --- | --- | --- |
|  | Left | Right | NIL |  |
| Right hand offered | Radio button | Radio button | Radio button |  |

a

Top of Form

| Top of Form  Bottom of Form | Top of Form  Bottom of Form  Paw given | | |  |
| --- | --- | --- | --- | --- |
|  | Left | Right | NIL |  |
| Left hand offered | Radio button | Radio button | Radio button |  |

35 Day 10

Top of Form

| Top of Form  Bottom of Form | Top of Form  Bottom of Form  Paw given | | |  |
| --- | --- | --- | --- | --- |
|  | Left | Right | NIL |  |
| Left hand offered | Radio button | Radio button | Radio button |  |

a

Top of Form

| Top of Form  Bottom of Form | Top of Form  Bottom of Form  Paw given | | |  |
| --- | --- | --- | --- | --- |
|  | Left | Right | NIL |  |
| Right hand offered | Radio button | Radio button | Radio button |  |

Once you have completed the Paw Task for this day, click 'Next' to move onto the Reach Task

p. 9 Task Two - Reach Task

Top of Form

Please complete this task at a different time in the day to the Paw Task

*Preferably when your dog is motivated to engage in play*

**Step 1 -** Find a piece of furniture with a gap. The gap should be large enough so that your dog can comfortably put his paw under

**Step 2 -** Find an object such as a favourite toy, slipper, teddy that your dog interacts with. Place the object in the gap. Ensure the object is far enough away so that the dog cannot reach for the object using their mouth (as shown in Figure 7). Whilst you are setting up, if possible, cue for your dog to sit/wait

**Step 3 -** Step away from the piece of furniture by around 1 metre and allow your dog to interact with the object. We ask you to move away from the furniture to reduce your presence causing bias in how the dog interacts.

**Step 4 -** Record which paw your dog uses first when reaching for the object in the following table. If they used their left paw (as shown in Figure 8), record this by clicking the 'Left' column. If they used their right paw (as shown in Figure 6), record this by clicking the 'Right’ column. If the dog used their mouth (as shown in Figure 7), please try again but put the object at a further distance. If the dog did not interact with the object after 60 seconds, record this as ‘NIL’.

**Step 5 -**Once the dog has reached for the object, or 60 seconds has passed, remove the object from under the furniture and allow your dog to play with it.

**This task should be repeated 10 times over a 28-day period when it is convenient for you and your dog. Once the task has been undertaken 10 times, no further trials are needed. Please use the same object for all trials, but if you must change object then please specify what object you changed to in the third column headed '***Any further notes***'.**

*If at any point your dog starts to show signs of frustration (e.g., raised hackles, barking, chasing tail), stop the trial.*

If you have any comments to add, in relation to how the trial went for you and your dog, please note them in the third column of the tables below.

Top of Form


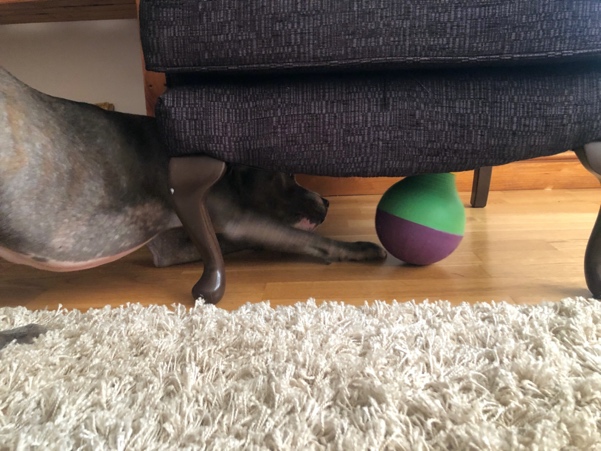

Example of dog reaching for object with their right paw in Reach Task


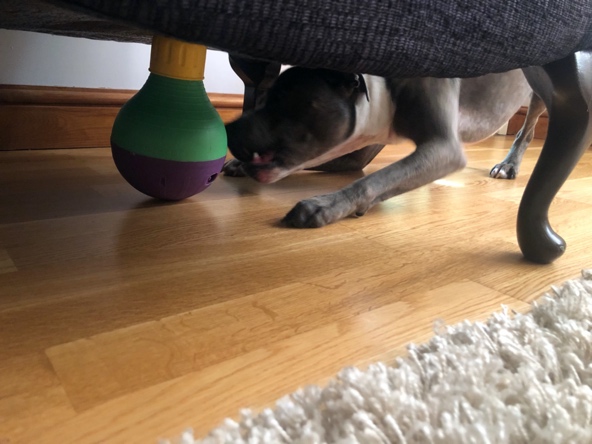

Example of dog reaching for object with no specific paw in Reach Task


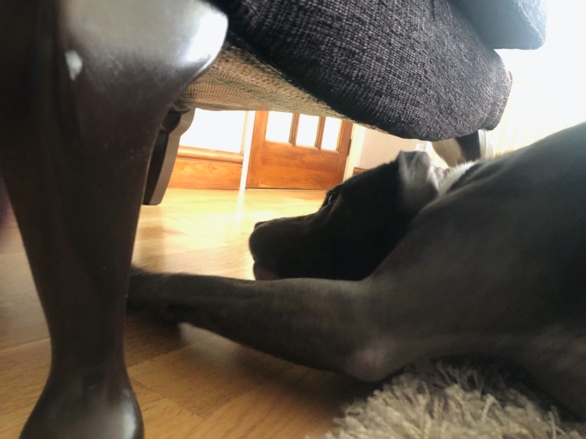

Example of dog reaching for object with their left paw in Reach Task

Top of Form

36 Please specify the object you will be using for the Reach Task:

Top of Form

37 Day 1

Top of Form

| Top of Form  Bottom of Form | Top of Form  First paw used to reach for object | | | Top of Form  Any further notes |  |
| --- | --- | --- | --- | --- | --- |
|  | Left | Right | NIL |  |  |
| Reach task | Radio button | Radio button | Radio button |  |  |

38 Day 2

Top of Form

| Top of Form  Bottom of Form | Top of Form  First paw used to reach for object | | | Top of Form  Any further notes |  |
| --- | --- | --- | --- | --- | --- |
|  | Left | Right | NIL |  |  |
| Reach task | Radio button | Radio button | Radio button |  |  |

39 Day 3

Top of Form

| Top of Form  Bottom of Form | Top of Form  First paw used to reach for object | | | Top of Form  Any further notes |  |
| --- | --- | --- | --- | --- | --- |
|  | Left | Right | NIL |  |  |
| Reach task | Radio button | Radio button | Radio button |  |  |

40 Day 4

Top of Form

| Top of Form  Bottom of Form | Top of Form  First paw used to reach for object | | | Top of Form  Any further notes |  |
| --- | --- | --- | --- | --- | --- |
|  | Left | Right | NIL |  |  |
| Reach task | Radio button | Radio button | Radio button |  |  |

41 Day 5

Top of Form

| Top of Form  Bottom of Form | Top of Form  First paw used to reach for object | | | Top of Form  Any further notes |  |
| --- | --- | --- | --- | --- | --- |
|  | Left | Right | NIL |  |  |
| Reach task | Radio button | Radio button | Radio button |  |  |

42 Day 6

| Top of Form  Bottom of Form | Top of Form  First paw used to reach for object | | | Top of Form  Any further notes |  |
| --- | --- | --- | --- | --- | --- |
|  | Left | Right | NIL |  |  |
| Reach task | Radio button | Radio button | Radio button |  |  |

43 Day 7

Top of Form

| Top of Form  Bottom of Form | Top of Form  First paw used to reach for object | | | Top of Form  Any further notes |  |
| --- | --- | --- | --- | --- | --- |
|  | Left | Right | NIL |  |  |
| Reach task | Radio button | Radio button | Radio button |  |  |

44 Day 8

Top of Form

| Top of Form  Bottom of Form | Top of Form  First paw used to reach for object | | | Top of Form  Any further notes |  |
| --- | --- | --- | --- | --- | --- |
|  | Left | Right | NIL |  |  |
| Reach task | Radio button | Radio button | Radio button |  |  |

45 Day 9

Top of Form

| Top of Form  Bottom of Form | Top of Form  First paw used to reach for object | | | Top of Form  Any further notes |  |
| --- | --- | --- | --- | --- | --- |
|  | Left | Right | NIL |  |  |
| Reach task | Radio button | Radio button | Radio button |  |  |

46 Day 10

Top of Form

| Top of Form  Bottom of Form | Top of Form  First paw used to reach for object | | | Top of Form  Any further notes |  |
| --- | --- | --- | --- | --- | --- |
|  | Left | Right | NIL |  |  |
| Reach task | Radio button | Radio button | Radio button |  |  |

If you have completed both the Paw Task and Reach Task for your Day, select 'Finish Later' so you can return to this page to complete the subsequent Day when you're next free

If you have **completed all** **10 Days** of Paw Task and Reach Task, please select **'Finish'**

Top of Form

p. 10 Final page

Top of Form

Thank-you for taking the time to participate in our study.

The data you have given us will be important for finding out whether owner handedness influences paw preferences in dogs.

If you would like to withdraw from the study, please get in touch with us using the emails below:
Kimberley C Charlton, 25227855@students.lincoln.ac.uk
Dr Elisa Frasnelli, efrasnelli@lincoln.ac.uk

If you are interested in the results of this study, a summary of this work can be found on:
Twitter: @UoLLifeSciences
Facebook: UoLLifeSciences

If you have another dog in the household and wish to take the study again then please follow this link:
[https://lincoln.onlinesurveys.ac.uk/owner-handedness-paw-preference-dogs](http://lincoln.onlinesurveys.ac.uk/owner-handedness-paw-preference-dogs)
